# The impact of a preoperative evaluation process on weight reduction and glycemic control in patients undergoing bariatric and metabolic surgery

**DOI:** 10.1002/osp4.735

**Published:** 2024-03-08

**Authors:** Jennifer Tempany, Andrew Collier, Abdulmajid Ali

**Affiliations:** ^1^ Bariatric Surgery Unit University Hospital Ayr Ayr UK; ^2^ School of Health & Life Sciences Glasgow Caledonian University Glasgow UK; ^3^ University of the West of Scotland Glasgow UK

**Keywords:** bariatric surgery, obesity, patient education, type 2 diabetes

## Abstract

**Introduction:**

Metabolic surgery is a sustainable intervention for obesity and type 2 diabetes. Preoperative education optimizes weight loss and glycemic control outcomes.

**Objective:**

This study aimed to determine the effect of a generalized preoperative evaluation process (PEP) in patients who underwent bariatric surgery on weight loss and glycemic control pre‐ and post‐surgery.

**Methods:**

Data were retrospectively collected and analyzed for patients with type 2 diabetes who underwent bariatric surgery between 2010 and 2016. Patients were categorized into two groups determined by participation in the PEP. The groups were named the PEP group and non‐PEP group. The correlation among engagement in the PEP was determined using the chi‐square test and *t*‐test. Statistical analysis with *p* < 0.05 was deemed significant.

**Results:**

129 patients were included in the study; 86 females (67%) and 43 males (33%). Fifty‐nine patients (46%) engaged in the PEP and 70 (54%) patients did not engage in the PEP. A greater reduction in weight loss was observed in the PEP group versus the non‐PEP group from initial enrollment to pre‐surgery (14.3 ± 9.2 kg vs. 11.6 ± 9.2 kg; *p* = 0.11), and from pre‐surgery to 2‐years post‐surgery (20.6 ± 14.8 kg vs. 16.9 ± 15.6 kg; *p* = 0.17). A greater reduction in HbA1c from initial enrollment to pre‐surgery was seen in the PEP group versus the non‐PEP group (0.90 ± 1.28% vs. 0.63 ± 1.07%); however, this was not maintained from pre‐surgery to 2‐year post‐surgery (0.51 ± 1.18% vs. 0.70 ± 1.73%). In both cases, the statistical difference was insignificant.

**Conclusion:**

The PEP was not associated with improvements in short‐term weight loss or glycemic control pre‐surgery and a 2‐years post‐surgery. Patients may benefit from individually tailored preoperative weight management strategies.

## Graphical abstract

This study aimed to determine the effect of a Pre‐operative Evaluation Process (PEP) in patients who underwent metabolic surgery between 2010 and 2016 on weight loss and glycemic control pre‐ and post‐surgery. PEP was not associated with improvements in short‐term weight loss or glycemic control pre‐surgery and a 2‐years post‐surgery.

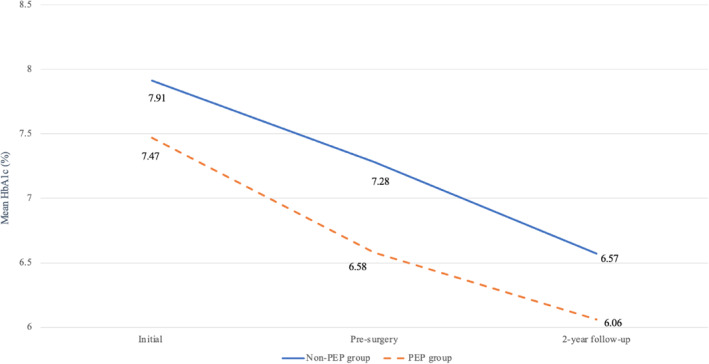

## 1 INTRODUCTION

The public health burden of obesity is extensive with the World Health Organization estimating that 13% of the adult population worldwide is overweight or obese.^1^ Obesity is a complex metabolic disorder underpinned by pathological alterations in genetic, neurobiological and hormonal pathways.^2^ Individuals with obesity are predisposed to metabolic syndrome, which is associated with a multitude of chronic diseases including type 2 diabetes, cardiovascular disease, non‐alcoholic fatty liver disease and a variety of cancers.^3^, ^4^, ^5^, ^6^ Bariatric and metabolic surgery can achieve long‐term weight reduction and improvements in glycemic control, resulting in metabolic changes which lead to improvements in insulin sensitivity and beta cell function.^7^, ^8^

Preoperative lifestyle interventions prior to bariatric and metabolic surgery are advocated by national guidelines including the American Society for Metabolic and Bariatric Surgery and the National Institute for Health and Care Excellence.^9^, ^10^ In preparation for bariatric and metabolic surgery, and to facilitate weight loss, patients frequently participate in targeted lifestyle interventions including physical activity programs, diet and behavioral modifications implemented by bariatric teams. There are wide variations in the structuring, content and endpoint of these programs, making it challenging to assess the impact on postoperative weight loss and glycemic control.^11^ On one hand, preoperative weight loss in patients undergoing bariatric and metabolic surgery has been associated with greater postoperative weight loss, shorter operating time and shorter length of hospital stay, thereby supporting the rationale for instigating preoperative weight management programs.^12^, ^13^, ^14^ On the other hand, drawbacks have been highlighted. Third‐party insurance companies often mandate a target weight loss of between 5% and 10% before insuring patients for surgery.^15^ Reaching this goal has been perceived as an additional barrier to obtaining surgery by both patients and clinicians.^16^ Failure to achieve this milestone could lead to delays in surgery, resulting in negative psychosocial consequences for the patient, thereby hindering their motivation and engagement in the overall process.^17^ Gregory et al. found that extended patient waits to surgery were associated with feelings of increased patient frustration and anger toward the preoperative process.^18^ As such, it remains unclear whether the preoperative weight management process serves to optimally prepare patients for the postoperative lifestyle changes required or simply act as a delay to necessary surgery.

In the NHS Ayrshire & Arran bariatric center, a resource intensive preoperative evaluation process (PEP) was introduced in July 2012 and was delivered by a multidisciplinary team involved in the patient's care. Prior to this, patients did not receive formal education in the pre‐surgery phase and qualified for bariatric and metabolic surgery if they had a BMI ≥40 kg/m^2^ or BMI ≥35 kg/m^2^ with an associated comorbidity. This study was undertaken to compare the effect of an intensive six session PEP in patients who underwent bariatric and metabolic surgery on weight loss and glycemic control pre‐ and post‐bariatric and metabolic surgery. The group who underwent the PEP was compared with the group of patients prior to July 2012 who did not undergo the PEP. Patients were therefore categorized into two groups based on whether they received preoperative education, and the outcomes were compared.

## 2 METHODS

Retrospective retrieval of anonymized data for patients with type 2 diabetes who underwent bariatric and metabolic surgery between 2010 and 2016 was undertaken. Patients were categorized into two groups determined by their participation in the PEP. The group who undertook the PEP included all patients who underwent formalized preoperative education and the non‐PEP group who did not participate in an education program. Patients who underwent bariatric and metabolic surgery were aged 18–65 years old, BMI ≥35 kg/m^2^ at the time of referral and had a diagnosis of type 2 diabetes. Bariatric and metabolic surgical procedures included in the study were laparoscopic sleeve gastrectomy and laparoscopic Roux‐en‐Y gastric bypass. Patients with insufficient data for 2‐year follow‐up and those who underwent revisional surgery were excluded.

The correlation among PEP compliance status and gender difference was determined using the chi‐square test and *t*‐test. IBM Statistical Package for the Social Sciences 23 statistical software was used for statistical analysis with *p* < 0.05 deemed statistically significant.

### 2.1 The PEP

The program consisted of six two‐hour sessions fortnightly over a 3‐month period encompassing a comprehensive multidisciplinary bariatric management program (Table 1). Patients were expected to attend all the sessions with a set target of 5% weight loss before surgery. The course was delivered by the bariatric multidisciplinary team consisting of surgeons, anesthetists, psychologists, physiotherapists, specialist nurses, dietitians, and patient representatives. Various topics were covered during the course.

**TABLE 1:** The PEP timetable.

Sessions	Topics covered	Facilitators
1	Diet and nutrition education	Bariatric dietitian
		Bariatric nurse
2	The Psychology of eating: emotional eating and coping strategies	Clinical psychologist
		Bariatric dietician
3	Practical cooking session	Bariatric dietitian
		Dietetic assistant practitioner
4	Surgery: surgical options, benefits and complications	Bariatric surgeon
	Anesthetics: anesthetic process	Anesthetist
		Endocrinologist
5	Physical activity regimes	Physiotherapist
	The Patient perspective: realistic expectations	Bariatric nurse
		Patient representatives
6	Long‐term behavior change and supplements following surgery	Bariatric nurse
		Bariatric dietitian

Abbreviation: PEP, Preoperative Evaluation Programme.

## 3 RESULTS

Between 2010 and 2016, 143 eligible patients with type 2 diabetes underwent bariatric and metabolic surgery in NHS Ayrshire & Arran. One hundred and twenty‐nine patients had complete data for 2‐years post‐surgery follow‐up and were included in the study. Of the 129 patients, there were 86 females (67%) and 43 males (33%); the mean age pre‐surgery was 49.8 ± 8.2 years old. The mean weight at initial enrollment, pre‐surgery, and 2‐year post‐surgery follow‐up was 131.9 ± 24.6, 119.1 ± 20.9, 100.5 ± 22.2 kg, respectively (Table 2).

**TABLE 2:** Baseline characteristics and reduction in weight (kg) and HbA_1c_ (%) of all patients at initial presentation, pre‐surgery and 2‐years post‐surgery (*n* = 129).

Characteristic	Initial presentation	Pre‐surgery	2‐years post‐surgery
Female, *n* (%)		86 (67)	
Age (years) mean ± SD		49.8 ± 8.2	
Age range (years)		28–63	
Weight, mean ± SD [range]	131.9 ± 24.6 [83.1, 225.6]	119.1 ± 20.9 [81.4, 186.6]	100.5 ± 22.2 [50.7, 171.4]
Decrease in weight (95% CI)	12.8 (11.2, 14.4) *p* < 0.001		
		18.6 (15.9, 21.3) *p* < 0.001	
HbA_1c_, mean ± SD [range]	7.71 ± 1.62 [5.2, 14.0]	6.96 ± 1.53 [4.8, 14.0]	6.34 ± 1.31 [4.6, 10.1]
Decrease in HbA_1c_ (95% CI)	0.75 [0.55, 0.96] *p* < 0.001		
		0.62 [0.35, 0.88] *p* < 0.001	

Abbreviations: CI, confidence intervals; HbA1c, glycated hemoglobin A1C; SD, standard deviation.

Fifty‐nine patients (46%) participated in the PEP. This group included 24 (41%) males and 35 (59%) females. Seventy (54%) patients did not participate in PEP, and of these patients there were 19 (27%) males and 51 (73%) females (Table 3). There was no statistical difference in gender (*p* = 0.10).

**TABLE 3:** Baseline characteristics of the PEP group and non‐PEP group patients at initial presentation, pre‐surgery and 2‐years post‐surgery (*n* = 129).

Characteristic	PEP group (*n* = 59)	Non‐PEP group (*n* = 70)
Female, *n* (%)	35 (59)	51 (73)
Age at surgery (years), mean ± SD	49.9 ± 8.0	49.7 ± 8.4
Age range (years)	33–63	28–62
Duration of diabetes (years), median (IQR)	4 (2–8.5)	5 (2–8)
Type of surgery		
LSG, *n* (%)	27 (45.8)	40 (57.1)
L‐RYGB, *n* (%)	32 (54.2)	30 (42.9)

Abbreviations: L‐RYGB, laparoscopic Roux‐en‐Y‐gastric bypass; LSG, laparoscopic sleeve gastrectomy; PEP, Preoperative Evaluation Program.

### 3.1 Weight reduction

**FIGURE 1:**
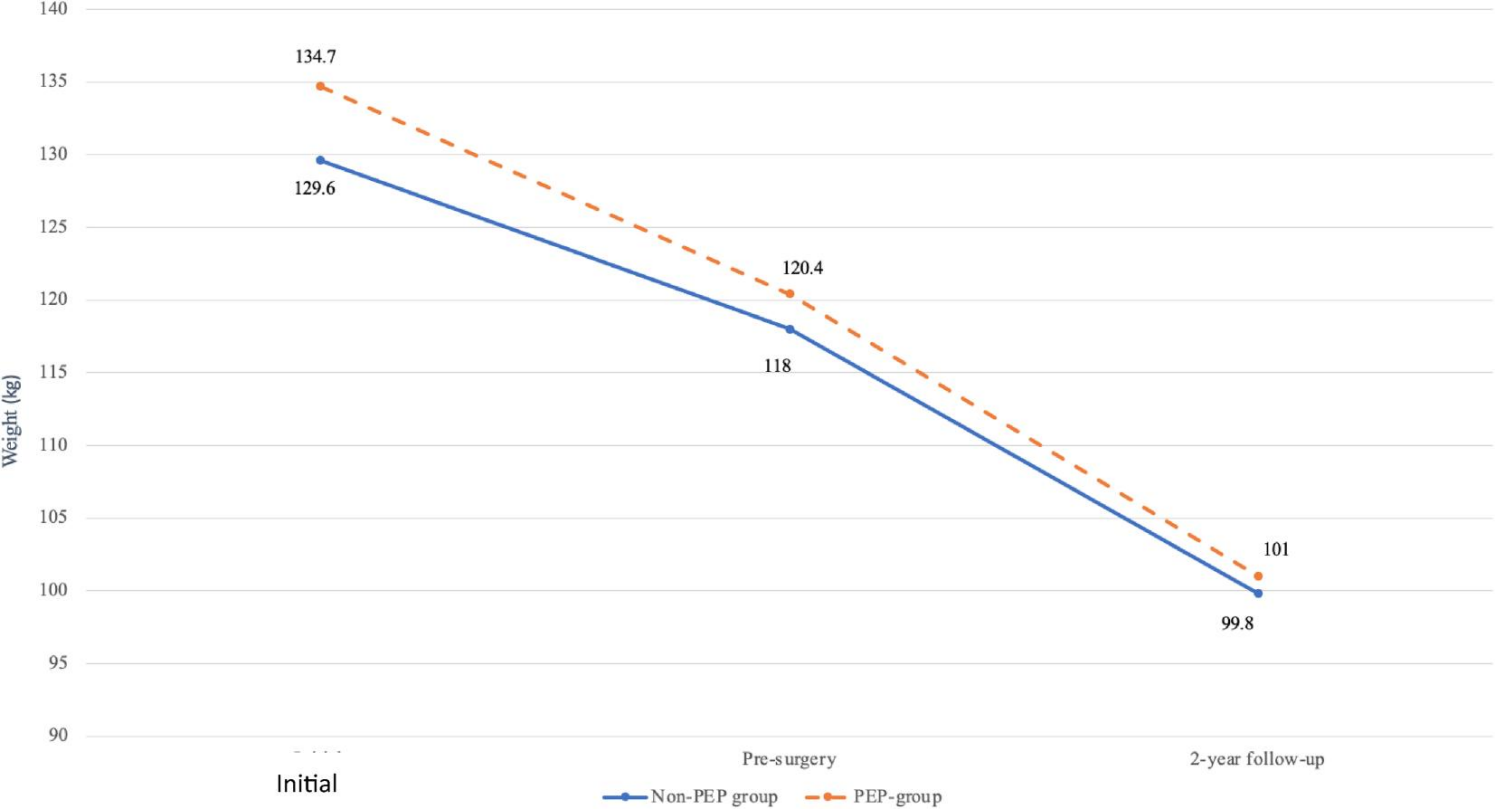
Comparison between the reduction in weight (kg) from initial enrollment to pre‐surgery and from pre‐surgery to 2‐years post‐surgery in the PEP group versus non‐PEP group. This figure shows a comparison in weight reduction between the PEP and non‐PEP groups at the time of initial enrollment to pre‐surgery and from pre‐surgery to 2‐year follow‐up. PEP, preoperative evaluation process.

There was a significant reduction in weight from initial enrollment to pre‐surgery and from pre‐surgery to 2‐year post‐surgery follow‐up in both groups. The mean reduction was 12.8 kg (*p* < 0.001) from initial enrollment to pre‐surgery and 18.6 kg (*p* < 0.001) from pre‐surgery to 2‐years post‐surgery, respectively. The group who undertook PEP achieved a greater reduction in weight from initial enrollment to pre‐surgery than the non‐PEP group (14.3 ± 9.2 kg vs. 11.6 ± 9.2 kg; *p* = 0.11), and a greater reduction in weight from pre‐surgery to 2‐year follow‐up (20.6 ± 14.8 kg vs. 16.9 ± 15.6 kg; *p* = 0.17). The differences, however, were not significant (Table 4). A comparison of weight loss between the two groups is shown in Figure 1.

**TABLE 4:** Reduction in weight (kg) and HbA_1c_ (%) from initial enrollment to pre‐surgery and from pre‐surgery to 2‐year post‐surgery follow‐up in PEP versus non‐PEP group (*n* = 129).

	PEP group (*n* = 59)	Non‐PEP group (*n* = 70)	Difference (*p*)
Decrease in weight from enrollment to pre‐surgery	14.3 ± 9.2	11.6 ± 9.2	2.7 NS *p* = 0.11
Decrease in weight from pre‐surgery to 2‐year follow‐up	20.6 ± 14.8	16.9 ± 15.6	3.7 NS *p* = 0.17
Decrease in HbA_1c_ from enrollment to pre‐surgery	0.90 ± 1.28	0.63 ± 1.07	0.27 NS *p* = 0.21
Decrease in HbA_1c_ from pre‐surgery to 2‐year follow‐up	0.51 ± 1.18	0.70 ± 1.73	−0.19 NS *p* = 0.47

Abbreviations: HbA1c, glycated hemoglobin A1C; PEP, Preoperative Evaluation Program.

### 3.2 Reduction in HbA_1c_ (%)

**FIGURE 2:**
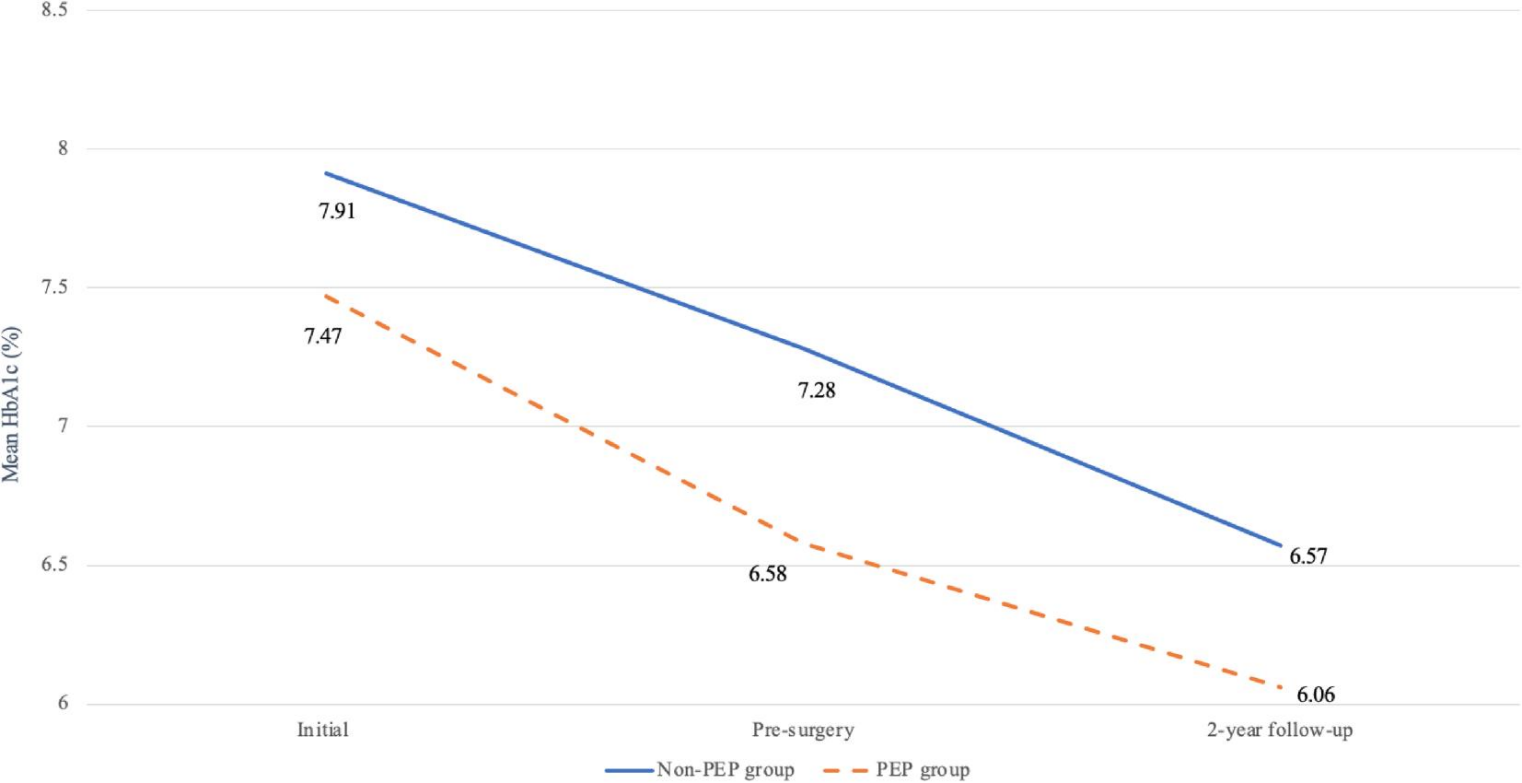
Comparison between the reduction in HbA_1c_ (%) from initial enrollment to pre‐surgery and from pre‐surgery to 2‐year follow‐up in the PEP group versus non‐PEP group. This figure shows a comparison in HbA_1c_ reduction between the PEP and non‐PEP groups at the time of initial enrollment to pre‐surgery and from pre‐surgery to 2‐year follow‐up. HbA1c, glycated hemoglobin A1C; PEP, preoperative evaluation process.

There was a significant reduction in HbA_1c_ from initial enrollment to pre‐surgery and from pre‐surgery to 2‐year follow‐up in both groups. The mean reduction in HbA_1c_ was 0.75% from initial enrollment to pre‐surgery and 0.62% % from pre‐surgery to 2‐year follow‐up, respectively. The group who undertook PEP achieved a greater reduction in HbA_1c_ from initial enrollment to pre‐surgery compared with the non‐PEP group (0.90 ± 1.28% vs. 0.63 ± 1.07%). This was not maintained from pre‐surgery to 2‐year follow‐up (0.51 ± 1.18% vs. 0.70 ± 1.73%). In both cases, the differences were not significant (Table 4). A comparison of the reduction in HbA_1c_ between the two groups is shown in Figure 2.

## 4 DISCUSSION

The authors of this study believed that altering behaviors prior to surgery would support patients in establishing the necessary modifications required postoperatively to optimize weight reduction. In this study, all patients, including those who participated in the PEP and those who did not, achieved a significant reduction in weight and glycemic control prior to surgery and at the 2‐year follow‐up (Table 2). However, there was no significant difference between the two groups at each stage, suggesting that the PEP had little impact on weight loss achieved at the time of surgery and at 2‐year follow‐up. Similar results were achieved in a recent meta‐analysis analyzing four studies, which indicated that preoperative lifestyle programs did not significantly impact on weight loss after 1‐year post‐surgery.^19^ This study also found that both groups achieved significant improvements in glycemic control from initial enrollment to pre‐surgery. Tight glycemic control preoperatively has been associated with reduced perioperative morbidity and mortality.^20^, ^21^, ^22^, ^23^

The group that undertook the PEP did achieve a non‐statistical greater weight loss pre‐surgery compared with the non‐PEP group (14.3 ± 9.2 kg vs. 11.6 ± 9.2 kg) and at 2‐year follow‐up (20.6 ± 14.8 kg vs. 16.9 ± 15.6 kg). Although speculative, the authors of this study suggest several potential explanations for this finding. The program followed a fixed structure with pre‐defined content, duration and frequency devised to educate on dietary changes, physical activity, and techniques to avoid emotional eating (Table 1). A variety of methods were used to deliver the sessions, ranging from small group lectures to one‐on‐one sessions. Patients undergoing bariatric surgery, however, have individualized needs with regard to behavioral changes and these may not have been addressed through a generalized program. A tailored weight management program constructed on an individual basis may be more applicable to patients preparing for bariatric and metabolic surgery and support the establishment of desired behaviors to assist patients in reducing their preoperative weight.^24^

Following referral for bariatric and metabolic surgery from 2012 onwards, in this study, all patients eligible for bariatric surgery were immediately enrolled into the PEP. Once the six sessions were completed, there were no further educational interventions prior to surgery. The length of time to surgery following completion of the PEP varied however, and this could have contributed to the lack of difference in weight loss between the two groups. Patients with prolonged delays to surgery due to waiting times may have felt demoralized and unmotivated. Delaying bariatric and metabolic surgery has been associated with increased patient anxiety and psychological burden, which is unlikely to support the formation of healthy lifestyle behaviors required for long‐term weight reduction.^25^ Furthermore, the lack of positive reinforcement and accountability for the change in habits learned on the PEP could have resulted in reduced implementation of these behaviors by patients prior to surgery, thereby reducing the overall impact of the PEP. Having a support group can help patients deal with the psychological stressors, dietary and behavior changes required for weight loss. A systematic review published in 2011 analyzing the effect of post‐surgical support groups following bariatric surgery found a positive association between engaging in support groups and weight loss.^26^ This was supported by a more recent study which found a positive correlation between the number of sessions attended in a post‐surgery support group and total percentage weight loss at 1 year.^27^ The authors of this study would suggest that structured education should be available post‐surgery and continue lifelong, speculating about that lifelong education would result in improved patient engagement leading to further loss of weight and improvements in glycemic control.

Implementing and sustaining preoperative educational programs is heavily resource intensive and can incur significant healthcare costs.^28^ This is in part due to the substantial coordination required between nurses, dieticians, surgeons and mental health professionals to sustain an educational program which tailors the range of lifestyle interventions to patient's needs. UK guidelines no longer require patients to achieve a target percentage weight loss prior to undergoing bariatric surgery; however, this is not the case in the US with many insurance companies necessitating patients to partake in a weight management program of a specific duration prior to authorizing insurance for surgery.^9^, ^29^ The PEP analyzed during the study involved a great deal of patient's time and was carried out in person meaning patients were required to travel to the hospital for attendance. Travel costs resulted in some patients not attending and therefore not completing the program highlight the healthcare inequalities associated with in‐person run weight management programs.

The retrospective nature of this study was a limitation. To undertake this study, the authors took advantage of the introduction of an education program in 2012, which allowed for a comparison of patients' weight and glycemic control. Patients in the group undergoing the PEP were weighted regularly, potentially introducing an element of selection bias particularly when accounting for patients excluded due to incomplete data. Of the 14 patients excluded, there were equal numbers from the group undertaking PEP and the non‐PEP group. Further studies are required to assess the quality of life in relation to preoperative weight management and education programs.

## 5 CONCLUSION

The preoperative patient education program in the NHS Ayrshire & Arran bariatric center, which is resource intensive, was not associated with improvements in short‐term weight loss or glycemic control.

## AUTHOR CONTRIBUTIONS

The study concept and design were contrived by Andrew Collier. The literature search and data retrieval were carried out by Jennifer Tempany. Analysis and interpretation of the data were undertaken by Jennifer Tempany, Andrew Collier and Abdulmajid Ali. The manuscript was drafted and revised by Jennifer Tempany, Andrew Collier and Abdulmajid Ali.

## CONFLICT OF INTEREST STATEMENT

The authors declare no conflicts of interest.
